# Non-Steroidal Anti-Inflammatory Drug Etoricoxib Facilitates the Application of Individualized Exercise Programs in Patients with Ankylosing Spondylitis

**DOI:** 10.3390/medicina56060270

**Published:** 2020-05-29

**Authors:** Iulia Rahela Marcu, Dalia Dop, Vlad Padureanu, Stefan Adrian Niculescu, Rodica Padureanu, Carmen Elena Niculescu, Otilia Constantina Rogoveanu

**Affiliations:** 1 Department of Physical and Rehabilitation Medicine, University of Medicine and Pharmacy of Craiova, 200349 Craiova, Romania; rmarcu@gmail.com (I.R.M.); otilia.rogoveanu@gmail.com (O.C.R.); 2 Department of Pediatrics, University of Medicine and Pharmacy of Craiova, 200349 Craiova, Romania; dalia_tastea@yahoo.com (D.D.); drcarmen88@yahoo.com (C.E.N.); 3 Department of Internal Medicine, University of Medicine and Pharmacy of Craiova, 200349 Craiova, Romania; vldpadureanu@yahoo.com; 4 Department of Orthopedics, University of Medicine and Pharmacy of Craiova, 200349 Craiova, Romania; niculescustefan94@gmail.com; 5 Department of Biochemistry, University of Medicine and Pharmacy of Craiova, 200349 Craiova, Romania

**Keywords:** ankylosing spondylitis, nonsteroidal anti-inflammatory drugs, physical exercise, quality of life

## Abstract

*Background and objectives:* The main objective of this study is to highlight the efficiency of different therapeutic means in patients with ankylosing spondylitis, resulting in the improvement of their quality of life. *Materials and Methods:* We conducted a randomized, longitudinal, controlled trial on 92 patients with ankylosing spondylitis over a period of 6 years. Disease activity was assessed using the BASDAI (Bath Ankylosing Spondylitis Disease Activity Index) score. The assessment of functional disabilities was performed using BASFI (Bath Ankylosing Spondylitis Functional Index). We assessed the quality of life using the HAQ questionnaire (Health Assessment Questionnaire). Based on the HAQ, we calculated the minimum number of patients to be treated for 52 weeks to prevent a decrease in the quality of life for at least one of them (the number needed to treat (NNT)). *Results:* For the combination therapy group, the result we obtained was 2, lower than the other therapies compared (the medication group and the group with physical exercise). We point out a correlation between the improvement of the functional status (BASFI) and the increase of the quality of life (HAQ), estimated as moderately high (0.8). The superiority of the effects of the combined treatment, in which we combined a nonsteroidal anti-inflammatory drug (etoricoxib) to the exercise program, is reflected by the model of the significant improvements (*p* < 0.05) obtained for the functional status and quality of life scores (BASFI and HAQ). *Conclusions:* The nonsteroidal anti-inflammatory drugs, in our case, etoricoxib, facilitate the application of individualized exercise programs in patients with ankylosing spondylitis.

## 1. Introduction

In the few last years, at an international level, there has been an increase in the number of cases diagnosed with ankylosing spondylitis (AS). AS is a chronic inflammatory disease, and its causes and mechanism of production are not yet clear [1]. Most likely, there is an involvement of environmental factors [2,3,4] that act on a predisposed genetic background, characterized by the presence of the HLA-B27 antigen [5,6]. Concomitantly, there is a continuous search for the best medication and rehabilitation treatments to slow down the inflammatory process [7,8]. 

Current drug treatment and diagnostic criteria are standardized through internationally developed therapeutic guidelines based on the observations and results of numerous clinical studies [9,10,11]. However, physical rehabilitation treatment, although a basic therapeutic treatment recommended for patients with ankylosing spondylitis, does not benefit from the same clear rules of application, and its effectiveness has not yet been correctly evaluated through extensive clinical studies [12]. By identifying the optimal exercise program to improve functional capacity, the results obtained will have substantial clinical and health implications for the patients with ankylosing spondylitis [13]. As with drug treatments, rehabilitation treatments practically target all the links of the pathogenic process of the disease by modulating reactivity, both immune and general [14].

The fundamental idea we relied on when formulating the objectives of this study is that the main purpose in the management of patients with ankylosing spondylitis is to ensure that these patients have a normal, fulfilled, and independent life. This fundamental objective translates to increasing joint mobility, muscular strength, and counteracting deformations and misalignments related to the periods of disease activity. 

We set out to develop an individualized rehabilitation program that will lead to an increase in the quality of life through family, social, and even professional integration of the patients, with a decrease in social and economic costs and to appreciate to what extent the results obtained are maintained after a year of treatment. We have developed a complex therapeutic program to include, in addition to a physical exercise program, other means of treatment (such as the administration of a nonsteroidal anti-inflammatory drug) and to compare the results of this treatment with those obtained in patients who only performed physical exercise.

## 2. Materials and Methods

### 2.1. Ethical Issues

This research was approved by the Academic and Scientific Ethics and Deontology Committee of the University of Medicine and Pharmacy in Craiova (Registration No. 36/2013) in accordance with the European Union Guidelines (Declaration of Helsinki). All the patients signed an information and acceptance form to be included in the present study.

### 2.2. Participants

We conducted a randomized, longitudinal, controlled trial on 92 patients with ankylosing spondylitis over 6 years between 2013 and 2019. Patients were monitored according to a unique research protocol and underwent complex periodical evaluations. The study aimed to highlight the efficiency of different therapeutic means, resulting in the improvement of quality of life.

- Inclusion criteria for the patients were as follows:
- patients’ participation agreement;
- patients diagnosed with ankylosing spondylitis according to the modified New York criteria;
- patients with axial forms of ankylosing spondylitis;
- no relevance was given to possible previous remissive therapies (including patients with discontinued treatment due to adverse effects or lack of therapeutic response) and to steroidal or nonsteroidal anti-inflammatory medication taken prior to the inclusion in the study.

Exclusion criteria for the patients were

- patients without axial forms of ankylosing spondylitis;
- patients in an inflammatory outbreak of the disease;
- heart failure or angina at rest or minimal effort;
- patients with silent coronary ischemia;
- blood pressure with difficult-to-control values;
- uncontrolled diabetes;
- chronic obstructive pulmonary disease (COPD);
- blood clotting disorders;
- cachexia;
- tumors in all evolutionary stages;
- active tuberculosis;
- fever;
- thrombophlebitis;
- severe mental disorders;
- pregnancy and breastfeeding period;
- alcohol addiction;
- non-cooperative patients;
- patients included in other ongoing clinical trials.

All patients met the modified New York criteria (1984).

From the clinical history of the patients, we followed

- demographic and anthropometric parameters: age, gender, place of origin, occupation;
- significant family history of inflammatory diseases: ankylosing spondylitis, psoriasis, sacroiliitis, uveitis;
- living and working conditions: nutrition, smoking, alcohol consumption, educational level, profession;
- the age of the patients at the onset of the disease and previous medication.

During the physical examination, we highlighted general condition, the temperature and weight curves, mobility of the spine and peripheral joints, muscular strength, assessment of functional status, and neuropsychiatric status. 

The clinical parameters for the patients were

- biological: acute phase reactants (ESR, CRP), HLA-B27 antigen, rheumatoid factor determination;
- imaging investigations: radiological examination of sacroiliac joints, the spine, and peripheral joints; nuclear magnetic resonance; musculoskeletal ultrasound.

### 2.3. Materials and Measures

Disease activity was assessed using the BASDAI (Bath Ankylosing Spondylitis Disease Activity Index) score. The assessment of functional disabilities was performed using the BASFI (Bath Ankylosing Spondylitis Functional Index) score. We assessed the quality of life using the HAQ questionnaire (Health Assessment Questionnaire). The monitoring of adverse reactions for the medication group was performed by hematological explorations (complete blood count) and biochemical determinations of serum aminotransferases and creatinine. 

Randomization was made in three groups, in order of inclusion.

- The group with medication, which followed only drug treatment throughout the study: etoricoxib 90 milligrams per day, together with hygiene and dietary measures (32 patients with ankylosing spondylitis—34.78%);
- the group with physical exercise, which followed a supervised, individualized exercise program for three months, then continued the exercises at home up to one year (24 patients with ankylosing spondylitis—26.08%);
- the group with medication and physical exercises (36 patients with ankylosing spondylitis—39.13%).

Patient monitoring was performed periodically, starting with the time of entry in the study (T1) and after 3 months, 6 months, and 1 year (T2, T3, and T4). The evaluation of the therapeutic efficiency was performed using clinical and paraclinical criteria and indices of evaluation of disease activity, functional status, and, especially, the quality of life. 

The drug that was administrated orally to the patients in the study was etoricoxib, 90 mg per day, an inhibitor of cyclooxygenase-2 (COX-2) of highly selective second-generation [15,16,17]. Etoricoxib has been successfully used for patients with osteoarthritis, rheumatoid arthritis, gout, and low back pain, and many clinical studies have highlighted its efficacy in patients with ankylosing spondylitis [18].

### 2.4. Statistical Analysis

All statistical analysis was performed with SPSS version 20 (IBM Corporation, Armonk, NY, USA). Descriptive statistics were used to map the characteristics using percentages for categorical variables and mean ± standard deviation (SD) for continuous variables. The groups were compared using the chi-square test for categorical variables and the Mann–Whitney U test for continuous variables. *p*-values less than 0.05 were considered statistically significant.

## 3. Results

The main purpose of the evaluation at the end of the study (T4) was to assess to what extent the results obtained after one year of treatment were maintained. 

**Figure 1:**
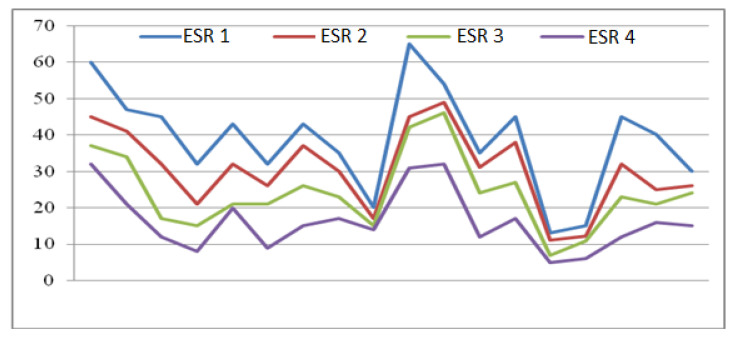
Evolution of erythrocyte sedimentation rate (ESR) in the moments T1–T2–T3–T4 in the etoricoxib and physical exercise group.

A first evaluation was the one regarding the markers of inflammation: erythrocyte sedimentation rate (ESR) and C-reactive protein (CRP). After one year, we noticed a sustained decrease in inflammatory markers that were significant in relation to the therapies (Table 1 and Figure 1). The most important decrease in inflammatory markers was obtained for the non-steroidal anti-inflammatory (NSAID) and exercise groups, and the results were statistically significant (*p* < 0.05).

**Figure 2:**
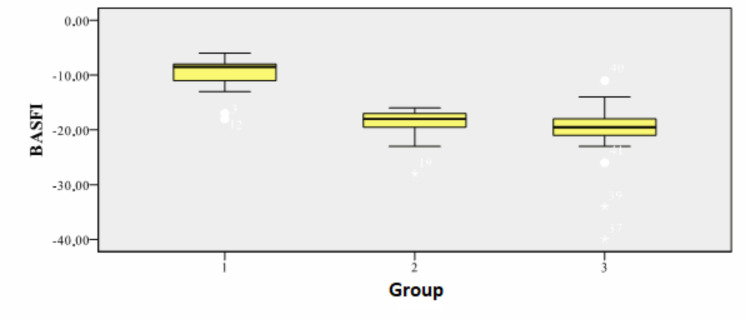
The difference BASFI 4 - BASFI 1 depending on the therapy.

To assess the effect of various therapies on the functional status of patients with ankylosing spondylitis, we calculated the difference between the average values of BASFI at the end of the study (T4) and those from the initial moment (T1). Under the effect of therapy, the decrease was differentiated (Figure 2).

The best result was obtained for Group 3, the one with physical exercise and etoricoxib. The differences between groups are highly significant statistically (*p* = 0.000008; Table 2).

The regression analysis applied to this indicator for the combination therapy group shows a possible decrease in the score of up to 39 points. This value, however, is dependent on the positive or negative influence of other factors, like the age of the patients, disease duration, and severity of the inflammatory process. 

**Figure 3:**
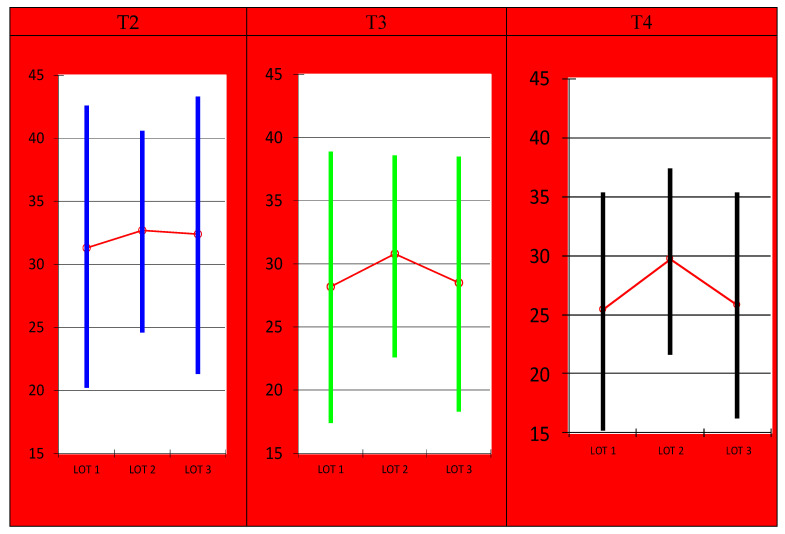
Evolution of the Bath Ankylosing Spondylitis Disease Activity Index (BASDAI) score.

Regarding the disease activity evaluated using the BASDAI score (Figure 3), the best results were registered in the study group with etoricoxib-only and the physical exercise program (on average, by 11,423 points), followed by the etoricoxib-only group, with an average decrease in the score of 9156 points, while the lowest decrease in the score was observed in the physical therapy group, on average, by 6190 points. The results were also statistically significant (*p* = 0.056981), proving the effectiveness of the combined treatment in influencing the activity of the disease.

**Figure 4:**
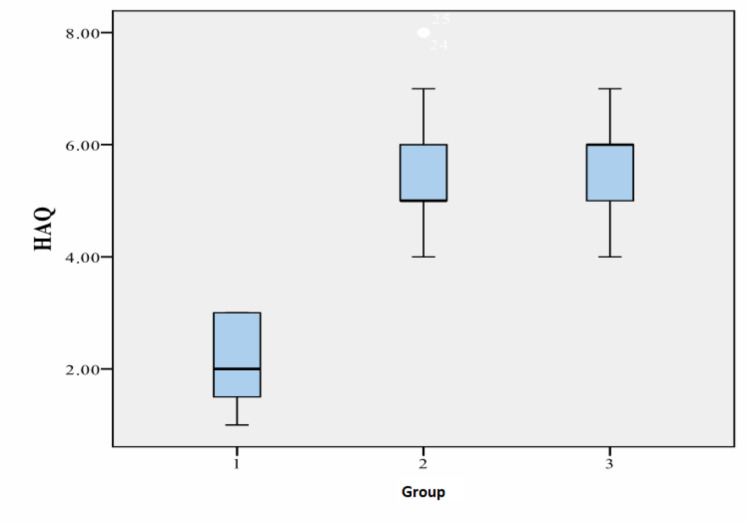
HAQ variation by therapy groups.

We considered it appropriate to evaluate the effectiveness of the therapy applied to patients through the evolution of the HAQ indicator; the assessment of each element of the scale is made with great discernment by the patients themselves. The dynamics of this indicator during 12 months of treatment reveal a more pronounced positive change in the combined therapy group (Table 3 and Figure 4).

Based on the HAQ indicator, we calculated the minimum number of patients to be treated for 52 weeks to prevent a decrease in the quality of life for at least one of them. We calculated

- **RAR**—the absolute reduction of the risk, which shows to what extent the risk of non-response decreases in the experimental group compared to the control group;
- **RRR**—the relative reduction of risk, which shows the benefit of the tested therapy as a percentage of the control;
- **NNT**—the number needed to treat, which is the number of patients who must benefit from comparative therapy for 12 months to prevent an adverse event that results in the deterioration of the patient’s quality of life due to the activity of the disease. The results are presented in Table 4.

The results highlight the impact of combined therapy on improving the quality of life of patients with ankylosing spondylitis. The minimum number of patients to be treated for 52 weeks to prevent a decrease in the quality of life for at least one of them is 2 in the group with combined therapy (etoricoxib and physical exercise program) and 3 in the group with the physical exercise program. 

For the whole study group, we observed the correlation of BASFI results with HAQ, with both parameters being sensitive in the evaluation of the physical function of the patients, and also the way in which the disease has an impact on their quality of life. 

Based on the results obtained, we can estimate that between BASFI and HAQ, there is an inverse, negative correlation, manifested not only at the level of the investigated groups but also at the population level of the patients with ankylosing spondylitis, whose size varies according to the applied therapy (Table 5). 

Sacroiliac joint (SI) damage induces low back pain that impacts physical function as it affects physical movement. In some cases, SI joint pain can be exacerbated by sports (as contact sports), regular heavy lifting, or labor-intensive jobs, while other activities as yoga and Pilates can be beneficial as they strengthen the joint ligaments. To maintain mobility and function is important to continue to engage in regular daily activities, to maintain good posture, to undertake regular exercise, which promotes strengthening and stretching. Physical therapy, low-impact exercise and massage, as well as thermotherapy, can stabilize and strengthen the SI joints and ease the pain. In the setting of inflammatory arthritis such as AS, anti-inflammatory medication is recommended to manage SI joint pain (Table 6). 

**Figure 5:**
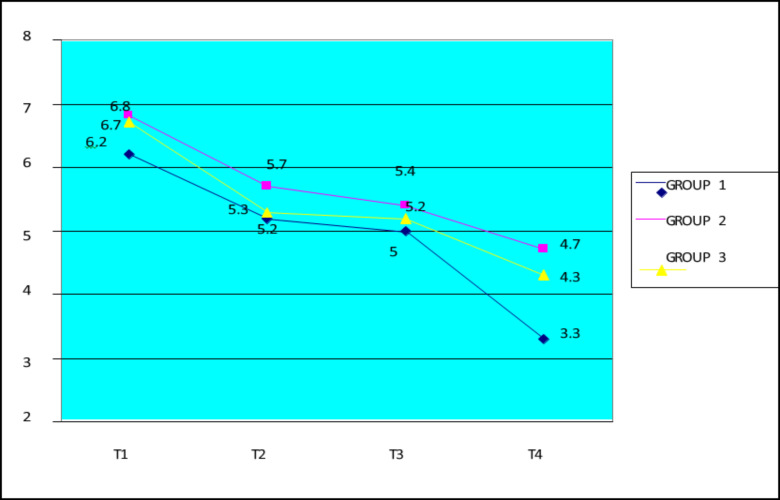
Visual analog scale (VAS) 1 dynamics in the three groups (nocturnal lumbosacral pain).

We evaluated SI pain by using a visual analog scale (VAS). Comparing the evolution of nocturnal lumbosacral pain evaluated on a visual analog scale (VAS1), we noticed that we obtained a decrease in pain symptomatology for all three groups. However, the best results were noted for the etoricoxib-only group (on average, by 2.913 points), followed by the study group with etoricoxib and physical exercise (on average, by 2.331 points), and by the physical-exercise-only group (on average, by 2.166 points) (Figure 5).

**Figure 6:**
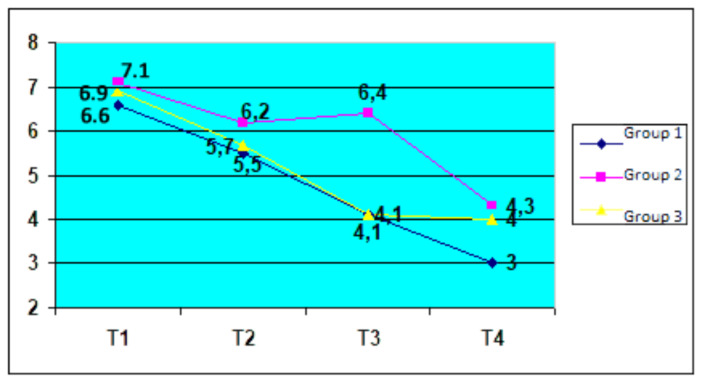
VAS 2 dynamics in the three groups with AS.

The same evolution was noticed for global lumbosacral pain, evaluated during the last week using VAS2 (a visual analog scale from 1 to 10). The most important decrease in pain was in the etoricoxib-only group (on average, by 3.558 points), followed by the study group with physical exercise and etoricoxib (on average, by 2.857 points). The least pain relief was recorded for patients in the exercise group (on average, by 2.778 points). The differences for these indicators were statistically significant (*p* < 0.05; Figure 6).

To evaluate the efficiency of the treatment, we monitored the dynamics of mobility in the peripheral joints (hip and knee). The best results were obtained for the study group with physical exercise and anti-inflammatory drug treatment. 

Thus for flexion of the hip joint, the combined treatment with etoricoxib and physical exercise determined an average increase of 21.667° between the initial and final evaluation moments, compared to the control groups (we obtained average increases of 19.667° for the physical exercise group and 11.563° for the etoricoxib-only group). For hip joint extension, the increase in mobility registered for the study group was, on average, 16.111° compared with the control groups (we noted an average increase of 12.5° for the physical exercise group and 5.625° for the etoricoxib-only group). 

The results obtained for hip abduction were as follows: an average increase of 20.00° for the combined therapy group, 15.00° for the physical exercise group, and 5.125° for the etoricoxib-only group.

Similar results were obtained for coxofemoral joint adduction: an average increase of 6.25° for the etoricoxib-only group and 12.916° for the exercise group; the best results were obtained for the study group with etoricoxib and exercise programs, with an average increase of 15.555°.

In this study, we also evaluated the rotation movements in coxofemoral joints. The mean values registered for the external rotation movement proved the efficiency of combined treatment applied to patients in the third group, where we obtained an average increase of 12.778° compared to the results obtained for the patients in the control groups (an average increase of 11.667° for patients with the physical exercise program and 5.313° for the etoricoxib-only group). The same results were noted for the internal rotation of the hip joint: an average increase of 4.062° for the etoricoxib-only group, 7.916° for the physical exercise group, and 9.444° for the study group with the etoricoxib and physical exercise program.

All results for hip joint mobility were highly significant statistically (*p* < 0.005). Because the inflammatory process leads, in time, to incomplete hip extension (flexion, and in compensation to knee flexion, we also evaluated knee mobility). The most significant decrease in knee flexion was obtained for patients in the same study group—9.444° compared to an average value of 7.583° in the physical exercise group and an average of 4.062° in the etoricoxib-only group. Table 7 shows the values for joint mobility after one year of therapy. We can notice that all differences are highly significant statistically (*p* < 0.05).

## 4. Discussion

Through combination therapy, the influence of BASFI in the improvement of HAQ is slightly higher than 50%, while the correlation between indicators is estimated as moderately increased (about 0.8).

Several categories of drugs with different therapeutic potential are included in the ankylosing spondylitis therapeutic arsenal [19]. One of the most significant and modern classification criteria divides these substances according to their effect on the evolution of the disease in

- SMARDs (symptom-modifying antirheumatic drugs), namely, NSAIDs and glucocorticosteroids [20,21];
- DMARDs (disease-modifying antirheumatic drugs), which include various substances used in the background treatment of these conditions, e.g., sulfasalazine, methotrexate, cyclophosphamide, leflunomide. Biological therapy is a more recent acquisition, offering new perspectives in the treatment of ankylosing spondylitis [22,23].

NSAIDs have indications in all forms of AS to control the inflammation that leads to the deterioration of the structures involved (pathogenic treatment) [24,25] and reduce the pain (symptomatic treatment) [26,27]. In patients with spondylitis, numerous studies have demonstrated the ability of NSAIDs to cause a significant improvement in inflammatory lumbar pain so that the response to NSAIDs is considered a useful diagnostic element [28]. 

Physical exercises are placed first in the hierarchy of forms of physical therapy and are applied immediately after solving the acute onset. Being an evolutionary disorder with a high degree of invalidity, the time to start physical exercise training must be as early as possible. The objectives of the physical exercise program are as follows: maintaining posture and body alignment, maintaining or increasing joint mobility, muscular strength, and prevention of restrictive respiratory dysfunction.

Suppressing spinal and peripheral inflammation, pain and stiffness, and muscular contracture, NSAIDs facilitate physical exercise [29].

## 5. Conclusions

To monitor the efficacy of the different therapeutic means in patients with AS, a complex initial evaluation is required from a clinical and functional point of view, and it is carried out using parameters that impact the quality of life. We found it appropriate to evaluate therapeutic efficacy with an instrument that reflects the patient’s point of view about what he thinks affects his quality of life, namely, the Health Assessment Questionnaire (HAQ).

NSAIDs represent the most common prescription in physical therapy practice. They increase the outcome of physical therapy treatments due to their beneficial effects. Many AS patients take NSAIDs before exercise programs to alleviate their pain [30]. Some clinical studies have determined that the short term use of NSAIDs can improve the recovery of muscle function [31]. There is a great number of clinical studies that demonstrate the beneficial effects of etoricoxib in AS [32].

The superiority of the effects of the combined treatment, in which we combined a nonsteroidal anti-inflammatory drug (etoricoxib) to the exercise program, is reflected by the model of the significant improvements (*p* < 0.05) obtained for the functional status and quality of life scores (BASFI and HAQ). The favorable evolution of the same parameters was noted in the patients from the control group with a physical exercise program, but the results were inferior to those obtained by the patients in the study group.

Based on HAQ, we calculated the minimum number of patients to be treated for 52 weeks to prevent a decrease in the quality of life for at least one of them (number needed to treat (NNT)). For the combination therapy group, the result we obtained was 2, which is lower than the other therapies compared (the etoricoxib-only group and the group with physical exercise).

The improvements recorded for the evaluated parameters have a strong resonance in the patient’s quality of life. Between therapy groups, the difference between the initial and final average values of the HAQ score is highly significant statistically (*p* < 0.001). We pointed out a correlation between the improvement of the functional status (BASFI) and the increase in the quality of life (HAQ), estimated to be moderately high (0.8).

We affirm the need for controlled, rigorous methodological studies to establish the optimal types of recovery treatments in patients with ankylosing spondylitis and the need to develop new evaluation methods.

## Figures and Tables

**Table 1:** Markers of inflammation after 12 months of therapy.

Variable at Time T4	Group			*p*-Value
**ESR**	Group	Mean	Standard Deviation (Std Dev)	0.0423661
	1	14,313	7867	
	2	19,833	9071	
	3	16,333	7282	
**CRP**	Group	Mean	Standard Deviation	0.041156
	1	7000	3817	
	2	7250	3372	
	3	5722	2322	

Group 1 etoricoxib only (N = 32), Group 2 physical exercise (N = 26), Group 3 etoricoxib and physical exercise (N = 36).

**Table 2:** Decrease of functional index Bath Ankylosing Spondylitis Functional Index (BASFI) during one year of treatment.

Group	Mean	Std Dev	Minimum	Median	75%ile	Maximum
1	−9938	3454	−18,000	−9000	−8000	−6000
2	−19,000	3384	−28,000	−18,000	−17,000	−16,000
3	−20,889	6781	−40,000	−19,500	−18,000	−11,000

**Table 3:** Health Assessment Questionnaire (HAQ)4–HAQ1.

Group	Mean ± Std Dev
1	2.000 ± 0.730
2	5.500 ± 1.382
3	5.611 ± 0.979

*p*-value = 0.000000.

**Table 4:** Patients percentage for RAR, RRR, NNT.

Treatment Applied and Monitored for 52 Weeks (Positive Event: HAQ Improvement)	RAR (How Much the Improvement Rate in the Experimental Group Increases Compared to the Control Group)	RRR (the Relative Benefit of the Studied Treatment)	NNT (the Minimum Number of Patients to be Treated for 52 Weeks to Prevent a Decrease in Quality of Life for at Least one of Them)
The physical exercise group	18%	40%	3
The etoricoxib and physical exercise group	27%	60%	2

**Table 5:** Correlation of BASFI and HAQ to the therapeutic protocol applied to patients with ankylosing spondylitis for 12 months.

Correlated Variables (Pearson Correlation for BASFI_i_ and HAQ_i_ at the Moment T_i_)	Studied Groups (the Meaning of the Correlation: +/−)		
	Group1: Etoricoxib Only	Group 2: with Physical Exercise	Group 3: with Etoricoxib and Physical Exercise
BASFI_1_ and HAQ_1_	−0.484 (*p* = 0.035)	−0.479 (*p* = 0.023)	−0.491 (*p* = 0.020)
BASFI_2_ and HAQ_2_	−0.511 (*p* = 0.002)	−0.448 (*p* = 0.000)	−0.570 (*p* = 0.000)
BASFI_3_ and HAQ_3_	−0.647 (*p* = 0.004)	−0.621 (*p* = 0.031)	−0.735 (*p* = 0.000)
BASFI_4_ and HAQ_4_	−0.650 (*p* = 0.006)	−0.626 (*p* = 0.039)	−0.789 (*p* = 0.000)

**Table 6:** Assessment of low back pain, disease activity, functional status, and quality of life after 12 months of treatment.

Variable at Moment T4	Group			*p*-Value
VAS1	Group	Mean	Std Dev	0.0449587
	1	3.275	2.029	
	2	4.667	0.888	
	3	4.333	1.249	
VAS2	Group	Mean	Std Dev	0.027745
	1	3.005	1.746	
	2	4.333	0.985	
	3	4.060	1.305	
BASDAI	Group	Mean	Std Dev	0.046981
	1	25.438	10,217	
	2	29.667	7.820	
	3	25.833	9.569	
BASFI	Group	Mean	Std Dev	0.041245
	1	42.750	16,591	
	2	40.667	15,144	
	3	37.611	15,209	
HAQ	Group	Mean	Std Dev	0.035688
	1	19.750	3.624	
	2	20.333	3.055	
	3	21.556	2.640	

Group 1 etoricoxib only (N = 32), Group 2 physical exercise (N = 26), Group 3 etoricoxib and physical exercise (N = 36) (the results represent mean ± std dev).

**Table 7:** The values for joint mobility after one year of therapy.

The Variable at Moment T4	Group			*p*-Value
FXCF4	Group	Mean	Std Dev	0.000452
	1	106.563	14.459	
	2	117.167	9.495	
	3	123.611	8.879	
EXCF4	Group	Mean	Std Dev	0.000148
	1	15.938	6.884	
	2	22.917	6.557	
	3	28.611	8.190	

Group 1 etoricoxib only (N = 32), Group 2 physical exercise (N = 26), Group 3 etoricoxib and physical exercise (N = 36).

## References

[1] Reveille J.D., Weisman M.H. (2013). The epidemiology of back pain, axial spondyloarthritis and HLA-B27 in the United States. Am. J. Med. Sci..

[2] Costello M.E., Elewaut D., Kenna T.J., Brown M.A. (2013). Microbes, the gut and ankylosing spondylitis. Arthritis Res. Ther..

[3] Ciccia F., Rizzo A., Triolo G. (2016). Subclinical gut inflammation in ankylosing spondylitis. Curr. Opin. Rheumatol..

[4] Schittenhelm R.B., Sian T.C., Wilmann P.G., Dudek N.L., Purcell A.W. (2015). Revisiting the arthritogenic peptide theory: Quantitative not qualitative changes in the peptide repertoire of HLA-B27 all types. Arthritis Rheumatol..

[5] Chen B., Li J., He C., Li D., Tong W., Zou Y., Xu W. (2017). Role of HLA-B27 in the pathogenesis of ankylosing spondylitis (Review). Mol. Med. Rep..

[6] Cortes A., Pulit S.L., Leo P.J., Pointon J.J., Robinson P.C., Weisman M.H., Ward M., Gensler L.S., Zhou X., Garchon H.J. (2015). Major histocompatibility complex associations of ankylosing spondylitis are complex and involve further epistasis with ERAP1. Nat. Commun..

[7] Taurog J.D., Chabra A., Colbert R.A. (2016). Ankylosing spondylitis and axial spondyloarthritis. N. Engl. J. Med..

[8] Wendling D., Lukas C., Prati C., Claudepierre P., Gossec L., Goupille P., Hudry C., Miceli-Richard C., Molto A., Pham T. (2018). 2018 update of French Society for Rheumatology (SFR) recommendations about the everyday management of patients with spondyloarthritis. Joint Bone Spine..

[9] Ward M.M., Deodhar A., Akl E.A., Lui A., Ermann J., Gensler L.S., Smith J.A., Borenstein D., Hiratzka J., Weiss P.F. (2016). American college of rheumatology/spondylitis association of America/spondyloarthritis research and treatment network 2015 recommendations for the treatment of ankylosing spondylitis and nonradiographic axial spondyloarthritis. Arthritis Rheumatol..

[10] National Institute for Health and Care Excellence (2017). Spondyloarthritis in Over 16s: Diagnosis and Management.

[11] Van der Heijde D., Ramiro S., Landewé R., Baraliakos X., Van den Bosch F., Sepriano A., Regel A., Ciurea A., Dagfinrud H., Dougados M. (2017). 2016 update of the ASAS-EULAR management recommendations for axial spondyloarthritis. Ann. Rheum. Dis..

[12] Levitova A., Hulejova H., Spiritovic M., Pavelka K., Senolt L., Husakova M. (2016). Clinical improvement and reduction in serum calprotectin levels after an intensive exercise programme for patients with ankylosing spondylitis and non-radiographic axial spondyloarthritis. Arthritis Res. Ther..

[13] Gladman D.D., Inman R.D., Cook R.J., Maksymowych W.P., Braun J., Davis J.C., Landewé R.B., Mease P., Brandt J., Vargas R.B. (2007). International spondyloarthritis interobserver reliability exercise—The INSPIRE study: II. Assessment of peripheral joints, enthesitis, and dactylitis. J. Rheumatol..

[14] Dagfinrud H., Kvien T.K., Hagen K.B. (2005). The Cochrane review of physiotherapy interventions for ankylosing spondylitis. J. Rheumatol..

[15] Agrawal N.G., Matthews C.Z., Mazenko R.S., Woolf E.J., Porras A.G., Chen X., Miller J.L., Michiels N., Wehling M., Schultz A. (2004). The effects of modifying in vivo cytochrome P450 3A (CYP3A) activity on etoricoxib pharmacokinetics and of etoricoxib administration on CYP3A activity. J. Clin. Pharmacol..

[16] Dallob A., Hawkey C.J., Greenberg H., Wight N., De Schepper P., Waldman S., Wong P., DeTora L., Gertz B., Agrawal N. (2003). Characterization of etoricoxib, a novel, selective COX-2 inhibitor. J. Clin. Pharmacol..

[17] Agrawal N.G., Rose M.J., Matthews C.Z., Woolf E.J., Porras A.G., Geer L.A., Larson P.J., Cote J., Dilzer S.C., Lasseter K.C. (2003). Pharmacokinetics of etoricoxib in patients with hepatic impairment. J. Clin. Pharmacol..

[18] Gossec L., van der Heijde D., Melian A., Krupa D.A., James M.K., Cavanaugh P.F., Reicin A.S., Dougados M. (2005). The efficacy of cyclooxygenase-2 inhibition by etoricoxib and naproxen on the axial manifestations of ankylosing spondylitis in the presence of peripheral arthritis. Ann. Rheum. Dis..

[19] Pedersen S.J., Sørensen I.J., Garnero P., Johansen J.S., Madsen O.R., Tvede N., Hansen M.S., Thamsborg G., Andersen L.S., Majgaard O. (2011). ASDAS, BASDAI and different treatment responses and their relation to biomarkers of inflammation, cartilage and bone turnover in patients with axial spondyloarthritis treated with TNFα inhibitors. Ann. Rheum. Dis..

[20] Birbara C.A., Puopolo A.D., Munoz D.R., Sheldon E.A., Mangione A., Bohidar N.R., Geba G.P. (2003). Etoricoxib Protocol 042 Study Group. Treatment of chronic low back pain with etoricoxib, a new cyclo-oxygenase-2 selective inhibitor: Improvement in pain and disability—A randomized, placebo-controlled, 3-month trial. J. Pain..

[21] Haibel H., Fendler C., Listing J., Callhoff J., Braun J., Sieper J. (2014). Efficacy of oral prednisolone in active ankylosing spondylitis: Results of a double-blind, randomised, placebo-controlled short-term trial. Ann. Rheum. Dis..

[22] Gao X., Wendling D., Botteman M.F., Carter J.A., Rao S., Cifaldi M. (2012). Clinical and economic burden of extra-articular manifestations in ankylosing spondylitis patients treated with anti-tumor necrosis factor agents. J. Med. Econ..

[23] Hebeisen M., Neuenschwander R., Scherer A., Exer P., Weber U., Tamborrini G., Micheroli R., Wildi L.M., Zufferey P., Nissen M.J. (2018). Response to tumor necrosis factor inhibition in male and female patients with ankylosing spondylitis: Data from a swiss cohort. J. Rheumatol..

[24] Wanders A., Heijde D., Landewé R., Béhier J.M., Calin A., Olivieri I., Zeidler H., Dougados M. (2005). Nonsteroidal anti-inflammatory drugs reduce radiographic progression in patients with ankylosing spondylitis: A randomized clinical trial. Arthritis Rheum..

[25] Sieper J., Listing J., Poddubnyy D., Song I.H., Hermann K.G., Callhoff J., Syrbe U., Braun J., Rudwaleit M. (2016). Effect of continuous versus on-demand treatment of ankylosing spondylitis with diclofenac over 2 years on radiographic progression of the spine: Results from a randomized multicenter trial (ENRADAS). Ann. Rheum. Dis..

[26] Hernandez-Garduno A., Vazquez-Leduc A., Querol-Vinagre J.V. Clinical evaluation following treatment with etoricoxib (60, 90 and 120mg once a day) in patients with acute low back pain: A cohort, open, non-randomized, multicenter study. Proceedings of the Annual Meeting of the European League Against Rheumatism.

[27] Pallay R.M., Seger W., Adler J.L., Ettlinger R.E., Quaidoo E.A., Lipetz R., O’Brien K., Mucciola L., Skalky C.S., Petruschke R.A. (2004). Etoricoxib reduced pain and disability and improved quality of life in patients with chronic low back pain: A 3 month, randomized, controlled trial. Scand. J. Rheumatol..

[28] Jansen J.P., Hunsche E., Choi E. Economic evaluation of etoricoxib versus non-selective NSAIDs in the treatment of ankylosing spondylitis in the UK [abstact]. Proceedings of the Annual Meeting of the European League Against Rheumatism.

[29] Jarret S.J., Mc Gonagle D., Marzo-Ortega H. Etoricoxib reduces the need for biologic therapy in ankylosing spondylitis (AS) but has no effect on magnetic resonance imaging. Proceedings of the American College of Rheumatology 68th Annual Scientific Meeting.

[30] Biederman R.E. (2005). Pharmacology in rehabilitation: Nonsteroidal anti-inflammatory agents. J. Orthop. Sports Phys. Ther..

[31] Lanier A.B. (2003). Use of nonsteriodal anti-inflammatory drugs following exercise reduced muscle injury. Sports Med..

[32] van der Heijde D., Baraf H.S., Ramos-Remus C., Calin A., Weaver A.L., Schiff M., James M., Markind J.E., Reicin A.S., Melian A. (2005). Evaluation of the Efficacy of Etoricoxib in Ankylosing Spondylitis: Results of a Fifty-Two-Week, Randomized, Controlled Study. Arthr. Rheum..

